# Exploration of the Quorum-Quenching Mechanism in *Pseudomonas nitroreducens* W-7 and Its Potential to Attenuate the Virulence of *Dickeya zeae* EC1

**DOI:** 10.3389/fmicb.2021.694161

**Published:** 2021-08-03

**Authors:** Wenping Zhang, Xinghui Fan, Jiayi Li, Tian Ye, Sandhya Mishra, Lianhui Zhang, Shaohua Chen

**Affiliations:** ^1^State Key Laboratory for Conservation and Utilization of Subtropical Agro-Bioresources, Guangdong Province Key Laboratory of Microbial Signals and Disease Control, Integrative Microbiology Research Centre, South China Agricultural University, Guangzhou, China; ^2^Guangdong Laboratory for Lingnan Modern Agriculture, Guangzhou, China

**Keywords:** *Pseudomonas nitroreducens* W-7, *N*-acyl homoserine lactones, quorum quenching, biocontrol, *Dickeya zeae* EC1

## Abstract

Quorum quenching (QQ) is a novel, promising strategy that opens up a new perspective for controlling quorum-sensing (QS)-mediated bacterial pathogens. QQ is performed by interfering with population-sensing systems, such as by the inhibition of signal synthesis, catalysis of degrading enzymes, and modification of signals. In many Gram-negative pathogenic bacteria, a class of chemically conserved signaling molecules named *N*-acyl homoserine lactones (AHLs) have been widely studied. AHLs are involved in the modulation of virulence factors in various bacterial pathogens including *Dickeya zeae*. *Dickeya zeae* is the causal agent of plant-rot disease of bananas, rice, maize, potatoes, etc., causing enormous economic losses of crops. In this study, a highly efficient AHL-degrading bacterial strain W-7 was isolated from activated-sludge samples and identified as *Pseudomonas nitroreducens*. Strain W-7 revealed a superior ability to degrade *N*-(3-oxododecanoyl)-l-homoserine lactone (OdDHL) and completely degraded 0.2 mmol/L of OdDHL within 48 h. Gas chromatography-mass spectrometry (GC-MS) identified *N*-cyclohexyl-propanamide as the main intermediate metabolite during AHL biodegradation. A metabolic pathway for AHL in strain W-7 was proposed based on the chemical structure of AHL and intermediate products. In addition to the degradation of OdDHL, this strain was also found to be capable of degrading a wide range of AHLs including *N*-(3-oxohexanoyl)-l-homoserine lactone (OHHL), *N*-(3-oxooctanoyl)-l-homoserine lactone (OOHL), and *N*-hexanoyl-l-homoserine lactone (HHL). Moreover, the application of strain W-7 as a biocontrol agent could substantially attenuate the soft rot caused by *D. zeae* EC1 to suppress tissue maceration in various host plants. Similarly, the application of crude enzymes of strain W-7 significantly reduced the disease incidence and severity in host plants. These original findings unveil the biochemical aspects of a highly efficient AHL-degrading bacterial isolate and provide useful agents that exhibit great potential for the control of infectious diseases caused by AHL-dependent bacterial pathogens.

## Introduction

*Dickeya zeae*, previously named *Erwinia chrysanthemi* pv. *zeae*, is a causal agent of plant-rot disease of dicotyledons and monocotyledons including bananas, rice, maize, and potatoes (Nassar et al., 1994; Hussain et al., 2008; Zhou et al., 2011; Hu et al., 2018). The occurrence and spread of *D. zeae*-associated diseases cause enormous economic losses of agricultural crops (Hussain et al., 2008; Pu et al., 2012). The constant outbreak of these diseases has received widespread attention and promoted research on the molecular aspects and pathogenic mechanisms of this emerging pathogen.

A range of virulence factors, including enzymes that degrade the extracellular plant cell wall (Hugouvieux-Cotte-Pattat et al., 2014), iron metabolism (Expert, 1999), siderophores (Franza et al., 2005), extracellular polysaccharides (Condemine et al., 1999), zeamine phytotoxins (Wu et al., 2010; Zhou et al., 2011; Cheng et al., 2013) in *D. zeae* EC1, the pigment indigoidine (Reverchon et al., 2002), and the type III secretion pathway in *Dickeya dadantii* 3937 (Yang et al., 2002; Yap et al., 2005), are strongly linked to the pathogenicity of *Dickeya* spp. and lead to plant diseases. The regulation of the virulence of *D. dadantii* and *D. zeae* is associated with two quorum-sensing (QS) systems (acyl homoserine lactones, AHL and Vfm; Nasser et al., 1998, 2013; Potrykus et al., 2018). Furthermore, several two-component system (TCS) proteins were found to be involved in the regulation of *D. dadantii* 3937’s pathogenicity, such as the PhoP-PhoQ TCS, regulating pectate lyase synthesis (Haque et al., 2005; Venkatesh et al., 2006). A type of GacS-GacA TCS modulates the expression of enzymes that degrade the plant cell wall (Lebeau et al., 2008). The VfmI-VfmH TCS is related to a *Dickeya*-specific quorum-sensing system (Nasser et al., 2013). In general, a complex regulatory network controls the production of various virulence factors in *Dickeya* spp. (Charkowski et al., 2012; Nasser et al., 2013; Reverchon and Nasser, 2013).

Concurrent with research into the molecular aspects and pathogenic mechanisms of *D. zeae* EC1, its genome has been sequenced (Zhou et al., 2015). The gene (*expI_Ecz_*) encoding AHL in *D. zeae* EC1 has been found, and its null mutation abolished AHL production, increased bacterial swimming and swarming motility, prevented the formation of multi-cell aggregates, eliminated the formation of cell aggregates in LB medium and partially attenuated the virulence of the pathogen in potato tubers (Hussain et al., 2008). In addition, it marginally reduced the inhibitory activity of *D. zeae* EC1 on rice-seed germination (Hussain et al., 2008). These results indicate that *D. zeae* EC1 possesses an AHL-type QS system, and the QS signal is essential for its modulation of cell motility and the regulation of bacterial virulence (Hussain et al., 2008). Furthermore, the TCS, the VfmI-VfmH signaling system, was also identified in the *D. zeae* strain EC1, which is involved in the regulation of cell wall-degrading enzymes, zeamine biosynthesis, cell motility, and pathogenicity (Lv et al., 2019).

The quorum quenching (QQ) mechanism has been found in many prokaryotes and eukaryotes, and its antibacterial activity is regulated by interfering with the communication of QS signals (Dong et al., 2007). The QQ strategy has opened up a new avenue of biocontrol and focuses on the control of pathogenic bacteria whose virulence factors are modulated by the QS system (Dong et al., 2002; Ye et al., 2019, 2020a; Wang et al., 2020). Several QQ genes have been identified, such as *aiiA* (the first) of *Bacillus* sp. (Dong et al., 2000), *aiiM* of *Microbacterium testaceum* (Wang et al., 2010), *attM* of *Agrobacterium fabrum* (Khan and Farrand, 2009), *ytnP* of *Bacillus subtilis* (Schneider et al., 2012), and *fadY* of *Acinetobacter lactucae* (Ye et al., 2020b). Previous research studies have demonstrated that bacteria or AHL-lactonase could reduce pathogenicity and protect hosts against Gram-negative pathogenic bacteria (Torres et al., 2016). The application of bacteria or AHL-lactonase to disrupt QS is being investigated in agriculture and could provide new opportunities for combating plant diseases caused by QS-modulated pathogenic bacteria.

The expression of the QQ enzyme AttM attenuated virulence in *Dickeya* spp. strains; soft-rot bacteria use AHLs as diffusible signals for coordinating QS communication (Crépin et al., 2012). This work points out that these key regulatory molecules appear to be credible targets for developing anti-virulence strategies against the *Dickeya* plant pathogens. Several strains, such as *Ochrobactrum intermedium* D-2, *Pseudomonas* sp. HS-18, *A. lactucae* QL-1, and *Cupriavidus* sp. HN-2, have revealed remarkable effects as biocontrol agents against *Xanthomonas campestris* pv. *campestris* and *Pectobacterium carotovorum* subsp. *carotovorum* in potato tubers, radishes, and their leaves (Ye et al., 2019, 2020a; Fan et al., 2020; Wang et al., 2020). These results illustrate that QQ strains have enormous potential for reducing the severity of the disease caused by QS-modulated pathogenic bacteria. However, there are few studies on the mechanisms of QQ related to AHL-degrading microorganisms for biocontrol against *D. zeae*.

In the present study, a novel, high-efficiency AHL-degrading strain, *Pseudomonas nitroreducens* W-7, was isolated and identified, and its AHL-degradation efficiency and mechanism of degradation were investigated. The properties of strain W-7 were explored to aid the development of biocontrol strategies against *D. zeae*. This preliminary study of strain W-7 as a biocontrol agent provides new insights for the control of AHL-dependent plant diseases.

## Materials and Methods

### Chemicals and Plants

Pure *N*-hexanoyl-l-homoserine lactone (C6HSL, HHL), *N*-(3-oxohexanoyl)-l-homoserine lactone (3OC6HSL, OHHL), *N*-(3-oxooctanoyl)-l-homoserine lactone (3OC8HSL, OOHL), and *N*-(3-oxododecanoyl)-l-homoserine lactone (3OC12HSL, OdDHL) were purchased from Shanghai UD Chem Technology Co., Ltd. Six antibiotics – ampicillin (Amp), chloramphenicol (Cm), tetracycline (Tc), streptomycin (Str), gentamicin (Gen), and kanamycin (Kan) – were employed for antimicrobial-susceptibility testing. Healthy plants, potato (*Solanum tuberosum* L.), radish (*Raphanus sativus* L.), Chinese cabbage [*Brassica pekinensis* (Lour.) Rupr.], and pakchoi (*Brassica campestris* L. ssp. *chinensis* Makino), were selected for the experiments and purchased from a local market (Guangzhou, China).

### Bacterial Strains and Media

The plant pathogen *D. zeae* EC1 was cultured in LB medium (NaCl, 10.0 g L^−1^; tryptone, 10.0 g L^−1^; and yeast extract, 5.0 g L^−1^) at 28°C and used in biocontrol assays (Fan et al., 2020). *Escherichia coli* DH5*α* was cultivated in LB medium at 37°C and used as a negative control in the biocontrol assay. *Agrobacterium tumefaciens* NT1 was applied as a biosensor to detect AHL. These strains were provided by the Integrative Microbiology Research Centre, South China Agricultural University, Guangzhou, China.

The mineral salt medium [MSM; (NH_4_)_2_ SO_4_, 2.0 g; Na_2_HPO_4_·12H_2_O, 1.5 g; KH_2_PO_4_, 1.5 g; MgSO_4_·7H_2_O, 0.2 g; CaCl_2_·2H_2_O, 0.01 g; FeSO_4_·7H_2_O, 0.001 g; 1,000 ml of H_2_O, and pH 6.5] and minimal medium [MM; (NH_4_)_2_SO_4_, 2.0 g; MgSO_4_·7H_2_O, 0.2 g; CaCl_2_, 0.01 g; FeSO_4_, 0.005 g; MnCl_2_, 0.002 g; K_2_HPO_4_, 10.5 g; KH_2_PO_4_, 4.5 g; mannitol, 2.0 g; glycerol, 2.0 g; 1,000 ml of H_2_O, and pH 6.5] were used in the biodegradation test and the determination of AHL-degrading metabolites.

### Isolation and Screening of QQ Strains

Bacterial strains were isolated by using an enrichment-culture technique (Chen et al., 2013; Huang et al., 2020). Activated sludge samples were collected from a sewer in South China Agricultural University, Guangzhou, China in March 2017 (113.356°E, 23.163°N). The OdDHL-degrading strains were enriched and isolated as previously described (Fan et al., 2020). The ability of the isolates to degrade OdDHL was evaluated using the biosensor strain *A. tumefaciens* NT1 as described in detail previously (Piper et al., 1993; Farrand et al., 2002; Steindler and Venturi, 2007; Fan et al., 2020). The pure isolate designated as W-7 showing the highest degradation activity was selected for further study.

### Identification of Strain W-7

The isolate W-7 was identified by genetic analysis based on 16S rDNA, morphology, and biochemical characteristics. Total genomic DNA was extracted using the EasyPure Bacteria Genomic DNA Kit (TransGen Biotech, Beijing, China). Two PCR primers, the forward primer (27F: 5ꞌ-AGAGTTTGATCCTGGCTCAG-3ꞌ) and reverse primer (1492R: 5ꞌ-GGTTACCTTGTTACGACTT-3ꞌ), were designed to amplify the 16S rDNA gene (Chen et al., 2013; Zhang et al., 2019). The reaction conditions consisted of initial denaturation at 94°C for 5 min 30 s, incubation at 55°C for 30 s, and extension at 72°C for 1 min 30 s, with the last cycle followed by 5-min extension at 72°C. The purified PCR product was sent to Shanghai Invitrogen Technology Co. Ltd., China, for sequencing. The resulting sequence was compared to sequences in GenBank using a BLAST search through the National Center for Biotechnology Information (NCBI). In addition, multilocus sequence analysis (MLSA) by using housekeeping genes DNA gyrase subunit B (*gyrB*), RNA polymerase *β* subunit (*rpoB*), and RNA polymerase subunit D (*rpoD*) for *Pseudomonas*, and using primer pairs of GyrBPUN1F/GyrBPUN1R, LAPS5F/LAPS27R, and PsEG30F/PsEG790R, respectively, is required to clarify the classification status of the W-7 strain (Li et al., 2020). All the resultant sequences were aligned and concatenated in the same order. Phylogenetic trees were constructed in MEGA version 5.0 using the neighbor-joining method (Yang et al., 2018; Zhan et al., 2018). Colony morphology was observed on LB plates incubated at 30°C for 24 h. An electron microscope (BH-2 Olympus, Japan) and scanning electron microscope (XL-30 ESEM; Philips Optoelectronics Co., Ltd., Holland) were used to investigate cellular morphologies of W-7.

### Antibiotic Sensitivity Test

An antibiotic susceptibility test was carried out on strain W-7 for further assays. A single colony of W-7 was inoculated into 1 ml of fresh LB medium and incubated overnight (12–16 h) at 30°C and 200 rpm. Amounts of 1 μl of cultures were inoculated into LB medium supplemented with different concentrations of various antibiotics and cultivated for 24 h at 30°C and 200 rpm. Six antibiotics (Cm, Amp, Str, Kan, Gen, and Tc) were used at different concentrations (5, 10, 15, 20, 50, 100, 150, and 200 μg ml^−1^). All treatments were carried out in triplicate and repeated three times.

### Biodegradation Test

To investigate the relationship between the growth of strain W-7 and OdDHL degradation, the assay was conducted in 250 ml flasks with 20 ml of MSM media supplemented with OdDHL (0.2 mmol L^−1^) and inoculated with an active-bacterial-cell suspension from the LB cultures. The non-inoculated flasks served as controls. The flasks were incubated at 30°C and 200 rpm on a rotary shaker for 2 days, and the OdDHL concentration was measured at 12 h intervals. The bacterial growth and OdDHL concentration were monitored using an UV spectrophotometer (at 600 nm, OD_600_) and high-performance liquid chromatography (HPLC) equipment, respectively. In addition, the abilities of strain XN-10 to degrade other AHLs (HHL, OHHL, and OOHL) were also determined. Samples of different treatments were collected, and degradation was performed as described above.

The residual quantity of AHL was determined by HPLC (Waters e2695, United States) under the following conditions: a detection wavelength of 210 nm, injection quantity of 20 μl, XBridge C_18_ reverse chromatographic column, flow rate of 0.8 ml min^−1^, column temperature of 30°C, and mobile phase of methanol/water = 65:35. It is performed under HPLC isocratic elution.

### Effect of Strain W-7 Against Soft Rot Caused by *Dickeya zeae* EC1

To investigate the potential ability of W-7 to control EC1, an *in vitro* assay was performed as described (Ye et al., 2019; Fan et al., 2020). The AHL-degrading W-7 bacterial isolates were tested for their ability to attenuate tissue maceration on host plants by EC1. A well-known AHL-degrading strain *Bacillus thuringiensis* subsp. *israelensis* B23 and agricultural streptomycin were used as a positive control, and a non-AHL-degrading strain *E. coli* DH5*α* was used as a negative control. Briefly, the host plants were washed and cut into 0.5 cm-thick slices after surface sterilization in 44% sodium hypochlorite and 75% ethyl alcohol. Cell PBS-suspension liquid (OD_600_ = 1.0) of *E. coli* DH5*α*, B23, W-7, and EC1 was inoculated on host plants washed three times with sterile water. Five treatments included pure EC1, EC1 mixed with *E. coli* DH5*α*, EC1 mixed with B23, EC1 mixed with agricultural streptomycin, and EC1 mixed with W-7. Five replications were used for each treatment, and each experiment was repeated four times. After inoculation, the host plants were placed in pallets and incubated at 28°C for 24 h. In order to express the disease severity numerically, the diameter and weight of the rot tissues were quantified to calculate the proportions in comparison to tissues only inoculated with EC1.

### Identification of AHL Metabolic Products

The overnight LB culture of strain W-7 (OD_600_ = 1.0–1.5) was centrifuged at 10,000 rpm for 1 min, and the supernatant was discarded. The cell pellets were washed twice with PBS buffer (0.01 M) and resuspended in MSM (pH 6.5) supplemented with HHL lactone at a final concentration of 1 mmol L^−1^. Samples were collected after cultivation at 30°C and 200 rpm for 8, 16, and 24 h, respectively. The residual AHL of the samples was extracted according to the method described in previous studies (Zhang et al., 2002; Wang et al., 2004; Fan et al., 2020). Three replicates were prepared for each treatment.

The metabolites were identified by gas chromatography-mass spectrometry (GC-MS; Agilent 6890N/5975, United States) analysis and matched with authentic standard compounds from the National Institute of Standards and Technology (NIST, United States) library database to determine the AHL-degradation metabolism. In the detection process, the temperatures of the injector, ion source, GC-MS interface, and quadrupole were controlled at 200, 230, 280, and 150°C, respectively. The flow rate of the carrier gas (helium) was kept at 1 ml min^−1^. The heating procedure was as follows: the column was held at 150°C for 2 min, increased to 280°C at the rate of 25°C/min, and held for 3 min (Fan et al., 2020).

### Antagonistic Test of Strain W-7

Antagonistic interactions between isolate W-7 and pathogen EC1 were investigated on the LB plate, according to the method of Fan et al. (2020). LB agar plates containing pathogen EC1 were punched with a sterilized perforator, and 20 μl of the cultures of strain W-7 and the metabolite solution were injected into the holes. In addition, 20 μl of sterile water and methyl alcohol were injected into the holes on another LB agar plate as controls, respectively. These plates were cultivated at 30°C for 24 h, and were observed for inhibition zones in the plate. Treatments were prepared in triplicate.

### Effect of W-7 Crude Enzymes Against EC1

The crude enzymes of strain W-7 were prepared according to the method of Ye et al. (2019). The overnight LB culture of strain W-7 was centrifuged (10,000 rpm for 10 min at 4°C), and the supernatant was used as an extracellular enzymes. Cells were washed thrice with PBS (0.01 M) and re-suspended in PBS. The suspension of strain W-7 was disrupted by sonication at 4°C, and then the homogenate was centrifuged (10,000 rpm for 10 min at 4°C). The harvested supernatant was used as a source of intracellular enzymes. The crude enzymes of strain W-7 was applied against soft rot caused by *D. zeae* EC1. The experimental design included four treatments: (1) Plant slices treated with EC1 at 4 × 10^8^ CFU ml^−1^; (2) Plant slices treated with 1 ml extracellular enzymes of W-7 and EC1, at 4 × 10^8^ CFU ml^−1^; (4) Plant slices treated with 1 ml intracellular enzymes of strain W-7 and EC1, at 4 × 10^8^ CFU ml^−1^; and (4) plant slices treated with sterile water served as control (Ye et al., 2019). Three replicates were performed for each treatment. Disease severity was evaluated as described above.

### Re-Lactonization Assay

In order to verify whether strain W-7 inactivated AHL *via* lactonase, 0.2 M HCl was added to the mixture of cells, along with OdDHL in MSM after 24 h of incubation. Acidification was carried out to enhance the re-lactonization of opened lactone rings. The OdDHL of the acidified mixture was detected using a biosensor, as described above (Fan et al., 2020).

### Acylase Activity Test

In order to detect whether the AHL-degrading enzyme of strain W-7 has acylase activity, we performed an analysis with an acylase activity assay kit (Solarbio, Beijing Solarbio Science & Technology Co., Ltd., China). The overnight LB culture of strain W-7 (OD_600_ = 1.0–1.5) was centrifuged at 10,000 rpm for 1 min, and the supernatant was discarded. The cell pellets were washed twice with PBS buffer (0.01 M) and resuspended in MSM (pH 6.5) supplemented with AHL at a final concentration of 1 mmol L^−1^. The supernatant was centrifuged and collected after cultivation at 30°C and 200 rpm for 24 h. The 50 μl of supernatant was used to test the acylase activity of strain W-7. Distilled water and *N*-acetyltransferase were served as negative control and positive control, respectively. Acylase can catalyze the transfer of the acetyl group of acetyl-CoA to butanol and, at the same time, reduce 5,5ꞌ-dithiobis-(2-nitrobenzoic acid; DTNB) to generate 2-nitro-5-thiobenzoic acid (TNB). The TNB compound appears as yellow, with an absorption peak at 412 nm (Lin et al., 2003).

## Results

### Identification and Characterization of Strain W-7

After the isolation and screening of the AHL-degrading strains, 15 pure isolates able to grow with AHL as the sole carbon source were obtained through enrichment culture as the biosensor strains. One bacterial strain, W-7, presenting higher AHL degradation activity as shown in Supplementary Figure S1, was selected for further experimental study.

Colonies of isolate W-7 appeared faint yellow in color, small, round, translucent, and smooth with neat edges when grown on LB plates for 24 h (Supplementary Figures S2A,B). The bacterium was Gram-negative and rod-shaped (length: 1.0–1.5 μm; width 0.5–0.7 μm) with a polar flagellum, as observed under a scanning electron microscope (Supplementary Figure S2C). The biochemical characteristics of this isolate are presented in Table 1. It tested positive for catalase, oxidase, fluorochrome, and nitrate reductase and utilized *α*-d-glucose, citric acid, *α*-keto-glutaric acid, *γ*-amino-butryric acid, *α*-d-glucose, and nalidixic acid; it tested negative in an anaerobic test, hydrogen sulfide, for gelatin liquefaction, in a hemolysis assay, and for starch hydrolysis and was unable to utilize d-maltose, dextrin, stachyose, d-trehalose, gentiobiose, sucrose, d-cellobiose, and d-turanose (Supplementary Table S1).

**Table 1:** Biochemical characteristics of strain W-7.

Characteristics	Results	Characteristics	Results
Gram staining	-	Anaerobic test	-
Hydrogen sulfide	-	Catalase	+
Gelatin liquefaction	-	Oxidase	+
Hemolysis assay	-	Fluorochrome	+
Starch hydrolysis	-	Nitrate reduction	+

+, tested positive and -, tested negative.

**Figure 1:**
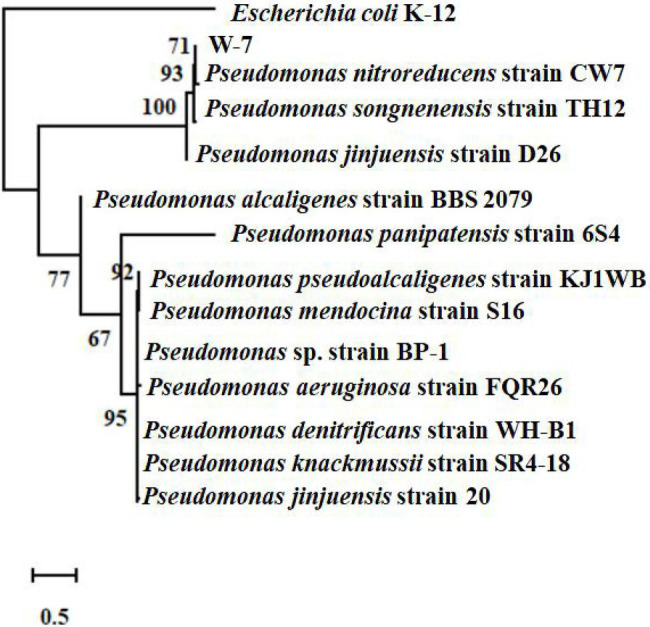
Phylogenetic tree based on the concatenated nucleotide sequences of the 16S rRNA, *gyrB*, *rpoB*, and *rpoD* genes of strain W-7. Consensus sequences of every gene from related bacterial strains were aligned with ClustalW and trimmed in the same sizes. All the sequences from the same strain were assembled to construct a joint neighbor-joining tree. Numbers in parentheses represent GenBank accession numbers. Numbers at the nodes indicate bootstrap values. Bar represents sequence divergence.

The 16S rRNA sequence of the isolate W-7 was shown to be close to that of *P. nitroreducens*. The sequence (1,411 bp) was submitted to GenBank under the accession number MG550968.1. MLSA analysis was performed based on the joint sequences of 16S rRNA, *gyrB*, *rpoB*, and *rpoD* for strain W-7. The joint phylogenetic tree indicated that strain W-7 was clustered together with *P. nitroreducens* (Figure 1). Based on morphological and biochemical characteristics and analysis of the multilocus sequence, the isolate was identified as *P. nitroreducens*.

The antimicrobial susceptibility of strain W-7 was tested to facilitate further assays. Supplementary Figure S3 shows the antibiotic sensitivity of *P. nitroreducens* strain W-7 to different antibiotics. Strain W-7 was able to withstand 400 mg ml^−1^ ampicillin (Amp); 50 mg ml^−1^ kanamycin (Kan); and 200 mg ml^−1^ gentamicin (Gen), streptomycin (Str), and chloromycetin (Cm). Strain W-7 exhibited resistance against tetracycline (Tc) up to 10 mg ml^−1^.

### Biodegradation Capacity of Strain W-7

**Figure 2:**
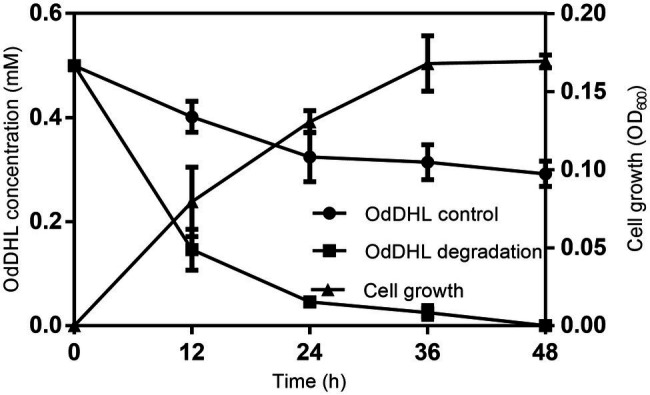
Degradation of *N*-(3-oxododecanoyl)-L-homoserine lactone (OdDHL) during the growth of strain W-7. Symbols: black rotundity ●, OdDHL control; black square ■, OdDHL degradation; black triangle ▲, growth of strain W-7.

**Figure 3:**
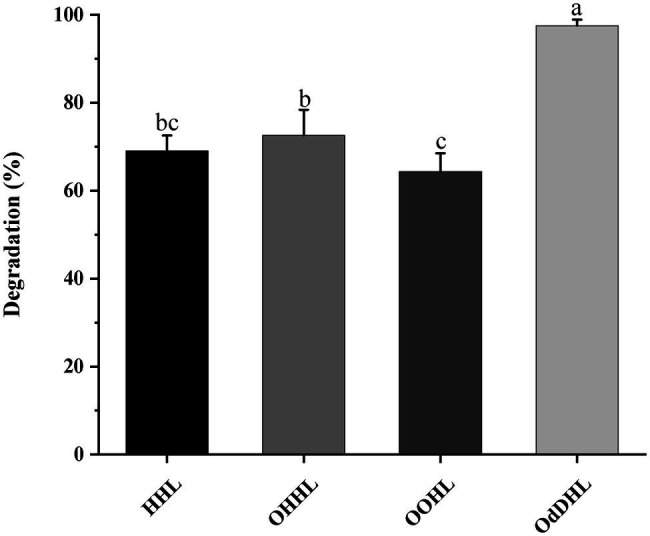
The ability of strain W-7 to degrade different acyl homoserine lactone (AHL) signal molecules. HHL, *N*-hexanoyl-l-homoserine lactone; OHHL, *N*-(3-oxohexanoyl)-l-homoserine lactone; OOHL, *N*-(3-oxooctanoyl)-l-homoserine lactone; and OdDHL, *N*-(3-oxododecanoyl)-l-homoserine lactone. Statistical analysis was performed by one-way ANOVA of Duncan method, and different letters indicate significant differences (*p* < 0.05) between treatments.

The growth-associated degradation of OdDHL was investigated in MSM medium under *in vitro* conditions. Samples at different intervals were collected for monitoring and recording the growth of strain W-7 and concentration of residual OdDHL. *Pseudomonas nitroreducens* W-7 utilized OdDHL (0.2 mmol L^−1^) as a sole carbon and energy source in MSM, as shown in Figure 2; under the conditions of 30°C and 200 rpm, 63.6% of the initial dose was rapidly degraded within 12 h. Finally, 0.2 mmol L^−1^ OdDHL was completely degraded after incubation for 48 h. The degradation of OdDHL by strain W-7 in MSM reached up to 63.6, 85.9, 91.9, and 100% after 12, 24, 36, and 48 h, respectively. The corresponding OD_600_ values were 0.054, 0.132, 0.162, and 0.165, respectively. These results show that bacterial growth increases as the OdDHL concentration decreases in the medium. The degradation of OdDHL increased during the exponential phase and decreased during the stationary growth phase. In addition to the degradation of OdDHL, this strain was also found to be capable of degrading a wide range of AHLs including OHHL, HHL, and OOHL, with a degradation efficiency of 72.6, 69.0, and 64.4%, respectively (Figure 3).

### Evaluation of Strain W-7 for Biocontrol of Soft-Rot Disease Caused by EC1

**Figure 4:**
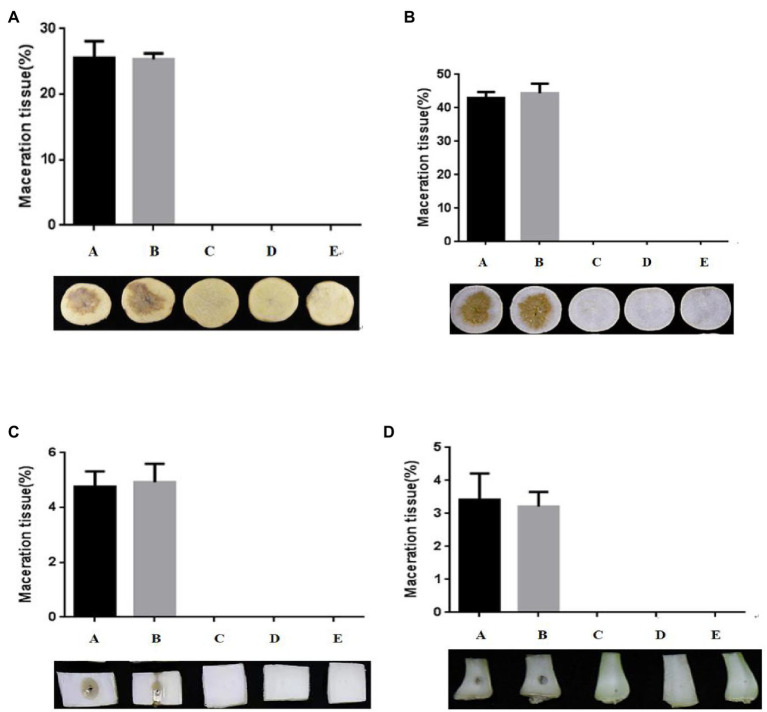
Test of strain W-7’s ability to attenuate maceration from soft-rot disease in plant slices. **(A)** potato (*Solanum tuberosum* L.); **(B)** radish (*Raphanus sativus* L.); **(C)** Chinese cabbage [*Brassica pekinensis* (Lour.) Rupr.]; **(D)** pakchoi (*Brassica campestris* L. ssp. *chinensis* Makino). Panel A, EC1 alone on plant slices; Panel B, EC1 + *Escherichia coli* DH5*α*; Panel C, EC1 + B23; Panel D, EC1 + agricultural streptomycin; Panel E, EC1 + W-7.

An *in vitro* biocontrol assay was carried out on potato (*Solanum tuberosum* L.; Figure 4A), radish (*Raphanus sativus* L.; Figure 4B), Chinese cabbage [*Brassica pekinensis* (Lour.) Rupr.; Figure 4C], and pakchoi (*Brassica campestris* L. ssp. *chinensis* Makino; Figure 4D) to assess the biocontrol capacity of strain W-7 against EC1. Strain B23, *E. coli* DH5*α*, and strain W-7 showed no pathogenicity toward potatoes in the inoculation experiment (data not shown). The results indicated that the soft rot only appeared on plant slices and stalks inoculated with EC1 and co-inoculated with *E. coli* DH5*α* and EC1. No soft-rot symptoms were observed on plant slices and stalks co-inoculated with strains EC1 and B23, or co-inoculated with agricultural streptomycin and EC1, similar to the treatment of strain EC1 and W-7. Furthermore, the degree of disease is quantitatively expressed by the macerated area (Supplementary Figures S4A–D). These results show that the macerated area and macerated tissue percentage of different treatment groups were consistent with the previous results. Strain W-7 significantly reduced the rotten-tissue area of strain EC1 on plant slices and stalks. These results indicate that the QQ strain W-7 is a potent biological control agent against the pathogen EC1 and may have the potential to be used to control other AHL-dependent bacterial pathogens.

### Biodegradation Products of Strain W-7

**Figure 5:**
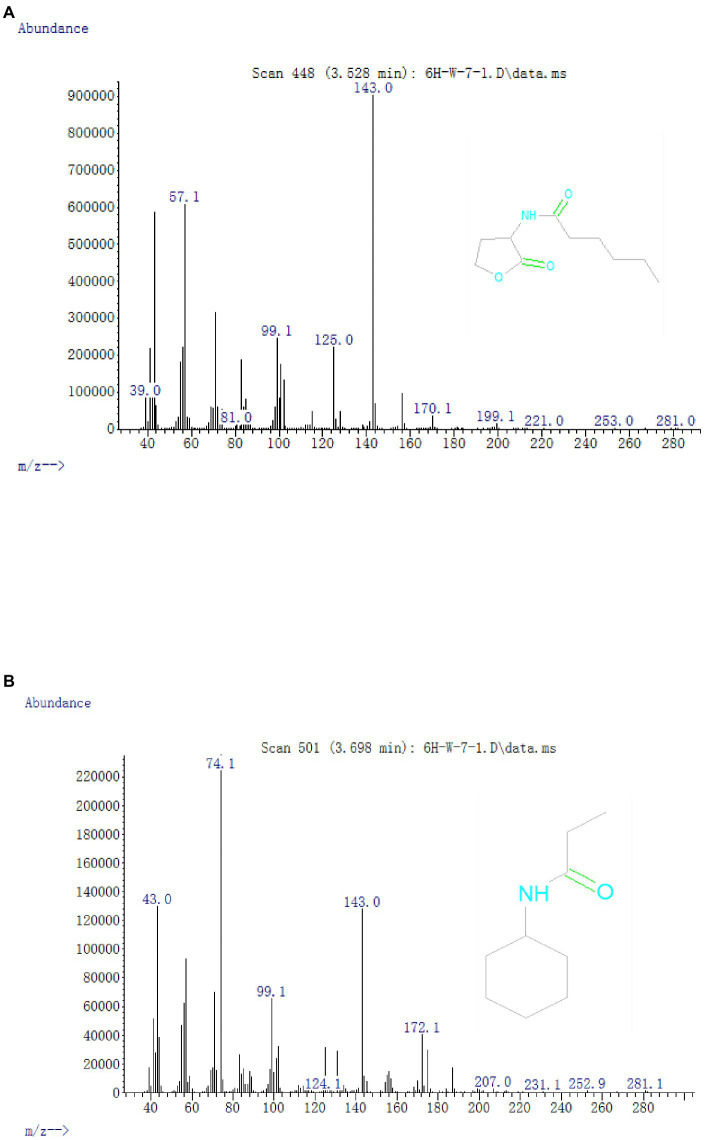
Mass spectra of AHL degradation products produced by strain W-7. **(A)** HHL; **(B)** *N*-cyclohexyl-propanamide.

To illuminate the AHL-degradation pathway, cells of strain W-7 were inoculated in MSM (pH 6.5) supplemented with AHL (final concentration, 1 mmol L^−1^). Samples were collected at 8, 16, and 24 h, and the degradation products were extracted, dried, and further resuspended in methanol and detected through GC-MS. The main products and HHL (Figure 5) were detected and identified from background-corrected mass spectra based on the similarity of their fragment retention times (RTs) and molecular ions for corresponding authentic compounds in the NIST library database.

**Figure 6:**
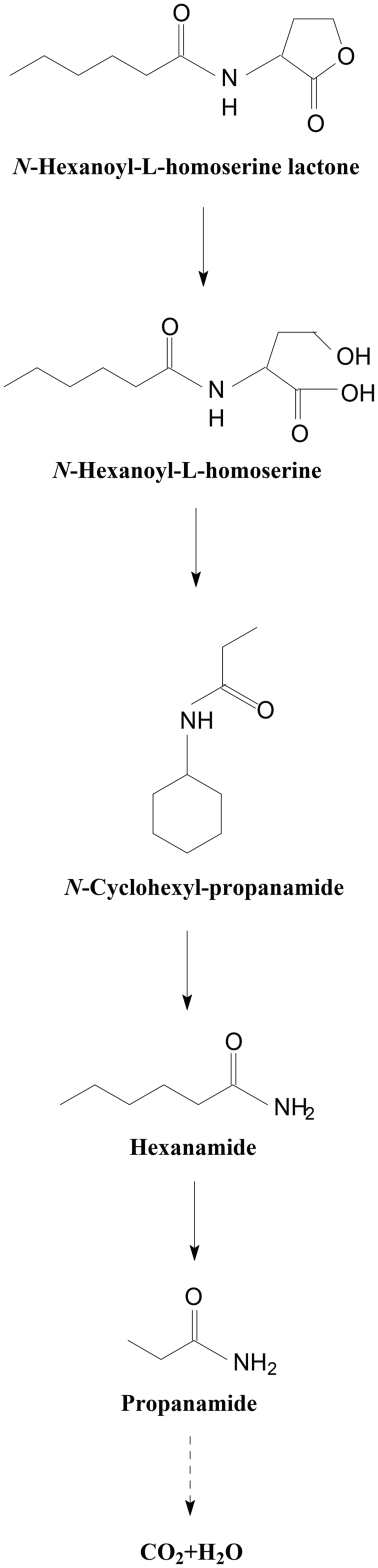
Proposed AHL-degradation pathway in strain W-7. HHL was initially degraded by the hydrolysis of its ester ring to yield *N*-hexanoyl-l-homoserine, which can be further degraded into *N*-cyclohexyl-propanamide by breaking the carbon-nitrogen bond, and then degraded to form hexanamide and propanamide. Finally, HHL was degraded into carbon dioxide and water without any persistent accumulative products.

In all the samples from 0 to 24 h, a significant compound was detected at 3.528 min, displayed a characteristic mass fragment [M+] at *m*/*z* = 143, and was identified as HHL (Figure 5A; Supplementary Figure S5A). HHL then gradually disappeared, with the formation of a new compound. This compound at the RT of 3.695 min showed a prominent protonated molecular ion at *m*/*z* 74 and was characterized as *N*-cyclohexyl-propanamide (Figure 5B; Supplementary Figure S5B). It is worth noting that the metabolites were transient and faded away at the end of the experiment. In this study, homoserine lactone (HSL) was not detected in the degradation process of AHL. Consequently, the AHL-degradation pathway in strain W-7 was proposed (Figure 6): HHL was initially degraded by the hydrolysis of its ester ring to yield *N*-hexanoyl-l-homoserine, which can be further degraded into *N*-cyclohexyl-propanamide by breaking the carbon-nitrogen bond, and then degraded to form hexanamide and propanamide. Finally, HHL was completely degraded without any persistent accumulative products.

### QQ Strain W-7 Did Not Show Antagonism Against EC1

To verify the antagonistic interactions between strain W-7 and pathogen EC1, bacterial suspension and metabolic extracts of strain W-7 were injected into the hole on the LB agar plate containing pathogen EC1. The results of the antagonism test showed that no inhibition zone was occurred when strain W-7 and pathogen EC1 grew together (Supplementary Figure S6). Therefore, these results confirmed that the W-7 strain showed no antagonism against pathogen EC1.

### Biocontrol Efficiency of W-7 Crude Enzymes

To evaluate the biocontrol effect of the crude enzymes of strain W-7, the *in vitro* biocontrol assay was carried out on potato tubers (Supplementary Figure S7A). The results showed that only slight decay was observed after treatment with intracellular and extracellular enzymes of strain W-7. Compared with the individual EC1 treatment group, the macerated area and the percentage of macerated tissues of the crude enzyme treatment group were significantly reduced (Supplementary Figure S7B). These results indicate that the crude enzymes of strain W-7 play a critical role in the biocontrol of soft rot caused by *D. zeae* EC1.

### Characterization of AHL Lactonase

To confirm whether the degradation of AHL by strain W-7 occurred by opening the lactone rings, a mixture of the QQ strain W-7 and OdDHL was incubated and acidified to reconstruct the lactonase cleavage site. The results showed that the OdDHL was degraded by strain W-7 and reconstructed after acidification (Supplementary Figure S8). The lactonase enzyme restored the structure of the lactone ring under acidic conditions. In addition, the acylase activity test for strain W-7 was negative (Supplementary Figure S9), suggesting that the degradation activity of strain W-7 is due to the presence of lactonase enzyme rather than acylase.

## Discussion

In this study, we isolated a novel AHL-degrading strain, *P. nitroreducens* W-7, from an activated-sludge sample collected from a sewer. *Dickeya zeae* is the causal agent of plant-rot disease of dicotyledons and monocotyledons, including bananas, rice, maize, and potatoes, and new hosts are being discovered (Nassar et al., 1994; Hussain et al., 2008; Zhou et al., 2011). Enormous economic losses have been brought about by *D. zeae*-associated diseases in agriculture (Hussain et al., 2008; Pu et al., 2012). We screened *P. nitroreducens* W-7, which could be considered as an efficient biocontrol agent, showing no pathogenicity toward crops (Figure 4). Strain W-7 could thus have potential as a safe agent for use in agriculture. Dong et al. (2004) found that *B. thuringiensis* B23 suppressed the quorum-sensing-dependent virulence of plant pathogen *Erwinia carotovora* through a new form of microbial antagonism, signal interference. *Bacillus thuringiensis* B23 significantly decreased the incidence of *E. carotovora* infection and symptom development of potato soft rot caused by the pathogen. Interestingly, similar effects were observed in *P. nitroreducens* strain W-7 in our studies. Moreover, application of strain W-7 and its crude enzymes as a biocontrol agent could substantially reduce the disease severity of EC1 on host plants. Furthermore, *P. nitroreducens* has been reported by other studies to have a strong ability to degrade diffusible signal factor (DSF; Wang et al., 2020) and other compounds, such as nicosulfuron (Zhao et al., 2018), chlorpyrifos (Aswathi et al., 2019), gamma-hexachlorocyclohexane (gamma-HCH; Zhang et al., 2010), *n*-hexanoyl homoserine lactone (Kaur and Yogalakshmi, 2020), and allethrin (Bhatt et al., 2020). However, few studies have reported the degradation of AHL-signaling molecules by *P. nitroreducens* (Kaur and Yogalakshmi, 2020).

Regarding the AHL-degradation ability of strain W-7, the results show that strain W-7 can completely degrade OdDHL signal molecules within 48 h. They also show potential degradation ability for other AHL signal molecules, such as HHL, OHHL, and OOHL. The isolated strain W-7 has better degradation ability for AHL signal molecules than the previously isolated AHL-degrading bacterium *Acinetobacter* sp. XN-10 (Zhang et al., 2020). At the same time, the metabolites and pathways of AHL degradation by *P. nitroreducens* W-7 were investigated. A new degradation pathway for AHL in strain W-7 is proposed as follows: AHL is initially degraded into *N*-hexanoyl-l-homoserine; then, *N*-hexanoyl-l-homoserine is converted into hexanamide and propanamide, and finally, propanamide is degraded into carbon dioxide and water.

Quorum quenching is regarded as a novel promising method for preventing and controlling bacterial diseases based on QS microorganisms, an alternative to antibiotics, to reduce the risk of drug resistance (Abed et al., 2013; Schuster et al., 2013; Rémy et al., 2020). There are various approaches to interfering with the QS system by QQ such as inhibiting signal synthesis, hindering the modification of signals, and inactivating QQ signals *via* enzymes or bacterial degradation (Bhatt et al., 2021). Among these, the use of QQ bacteria or QQ enzymes has appeared as an efficient tool for managing plant diseases (Uroz et al., 2009). Several QQ strains such as *Cupriavidus* sp., *A. lactucae*, *Burkholderia anthina*, *O. intermedium*, *Bacillus* sp., *Variovorax* sp., *Variovorax paradoxus*, and *A. tumefaciens* have been discovered and applied as tools for the biocontrol of plant diseases (Ha et al., 2018; Ye et al., 2019, 2020b,c; Fan et al., 2020). The expression of QQ hydrolytic enzymes (AttM and AiiA) quenched virulence in the pathogenic bacteria *Dickeya* spp. and *P. carotovorum* subsp. *carotovorum* (Dong et al., 2002; Crépin et al., 2012). However, in the case of *P. nitroreducens*, there are few studies on the QQ mechanisms (Kaur and Yogalakshmi, 2020).

*Dickeya zeae* EC1 is the most widely investigated strain of *D. zeae*, whose whole genome has been sequenced and published (Zhou et al., 2015). An AHL-type QS system was found in *D. zeae* EC1, and its QS signal is vital for the modulation of cell motility and bacterial virulence in EC1 (Hussain et al., 2008). Several TCS proteins are involved in the modulation of cell wall-degrading enzymes, zeamine biosynthesis, cell motility, and pathogenicity (Lv et al., 2019), such as the PhoP-PhoQ TCS in *D. dadantii* 3937, VfmI-VfmH TCS in the *Dickeya*-specific quorum-sensing system, and GacS-GacA TCS (Haque et al., 2005; Venkatesh et al., 2006; Lebeau et al., 2008; Nasser et al., 2013).

Strain EC1 is a QS-modulated bacterium, and its cell motility and virulence are regulated by the QS system. Theoretically, QQ bacteria can be employed as efficient biological tools for managing disease caused by strain EC1, but no strains have been discovered to fight against it. In a search for naturally occurring biological control agents, a novel bacterial strain *P. nitroreducens* W-7 from wastewater samples was screened for its biocontrol potential against EC1. The results demonstrated that strain W-7 effectively attenuated the maceration caused by the pathogen *D. zeae* EC1 in host plants. Similarly, the application of crude enzymes of strain W-7 substantially reduced the disease incidence and severity in host plants. These results reveal that strain W-7 and its enzymes have excellent potential for application as a biocontrol agent for preventing and controlling plant diseases caused by AHL-mediated pathogenic bacteria. In addition, based on the results of the antagonism test, it can be proved that the disease inhibitory activity of strain W-7 is not due to its own antimicrobial activity. The re-lactonization assay further confirmed that strain W-7 inactivated AHL *via* lactonase enzyme. In contrast to previous studies, HSL was not observed in the degradation process of AHL by strain W-7 (Lin et al., 2003). In addition, our results showed that the acylase activity test for strain W-7 was negative. Therefore, these results further demonstrated that the degradation activity of strain W-7 is due to the presence of lactonase enzyme rather than acylase. Hence, it can be inferred that the mechanism of strain W-7 disease suppression may be quorum quenching. This antagonism test is only indirect evidence. In the future, we will use a QQ-negative mutant of strain W-7 to conclusively demonstrate the function of QQ in disease suppression.

## Conclusion

This study identified a novel QQ candidate, *P. nitroreducens* strain W-7, to combat soft rot caused by *D. zeae* EC1. QQ strain W-7 possesses superior AHL-degradation ability and has the capacity to degrade a wide range of AHLs. Strain W-7 harbors the metabolic pathway for the complete degradation and metabolism of AHL. Moreover, application of strain W-7 and its crude enzymes as a biocontrol agent can substantially reduce the severity of the disease caused by *D. zeae* EC1 in host plants. This study provides a new option for the biocontrol of plant diseases and also lays a foundation for further research on the mechanisms of the inhibition of plant pathogens from the perspective of QQ. An exploration of the molecular mechanisms in further depth will provide insights into the biocontrol activity of strain W-7.

## Data Availability Statement

The original contributions presented in the study are included in the article/Supplementary Material, further inquiries can be directed to the corresponding author.

## Author Contributions

SC and LZ conceived the presented idea. WZ and XF contributed to the writing and prepared the figures and tables. JL, TY, SM, and SC participated in revising the manuscript. All authors contributed to the article and approved the submitted version.

## Conflict of Interest

The authors declare no conflict of interest. The funders had no role in the design of the study; in the collection, analyses, or interpretation of data; and in the writing of the manuscript, or in the decision to publish the results.

## Publisher’s Note

All claims expressed in this article are solely those of the authors and do not necessarily represent those of their affiliated organizations, or those of the publisher, the editors and the reviewers. Any product that may be evaluated in this article, or claim that may be made by its manufacturer, is not guaranteed or endorsed by the publisher.
