# Supplementary material for: Exploration of the Quorum-Quenching Mechanism in Pseudomonas nitroreducens W-7 and Its Potential to Attenuate the Virulence of Dickeya zeae EC1
Source: Front Microbiol. 2021 Aug 3;12:694161. doi: 10.3389/fmicb.2021.694161 (PMC8369503; doi:10.3389/fmicb.2021.694161)
Supplement: Supplementary file 1 [file Data_Sheet_1.docx]

Supplementary Material

**A B C D**


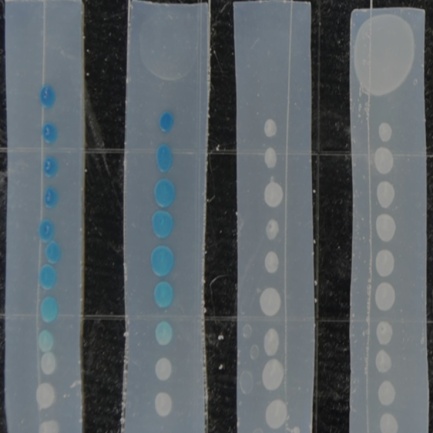


**Figure S1.** Degradation of OdDHL by strain W-7. A: negative control: only containing OdDHL (20 μmol·L^-1^). B: negative control: degradation of OdDHL (20 μmol·L^-1^) by *E. coli* DH5*α.* C: positive control: degradation of OdDHL (20 μmol·L^-1^) by *Bacillus thuringiensis* subsp. *israelensis* B23. D: degradation of OdDHL (20 μmol·L^1^) by strain W-7. The diffusion length of OdDHL and the blue colonies increase with the increase of OdDHL concentration. Each colony corresponds to the biosensor strain *Agrobacterium tumefaciens* NT1.


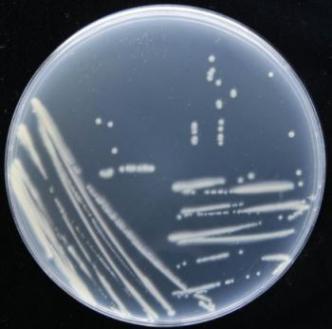

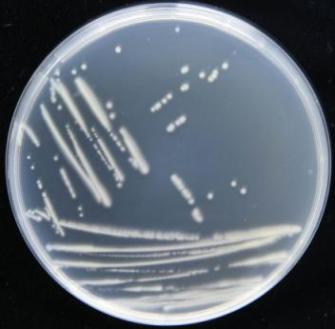


**(b)**


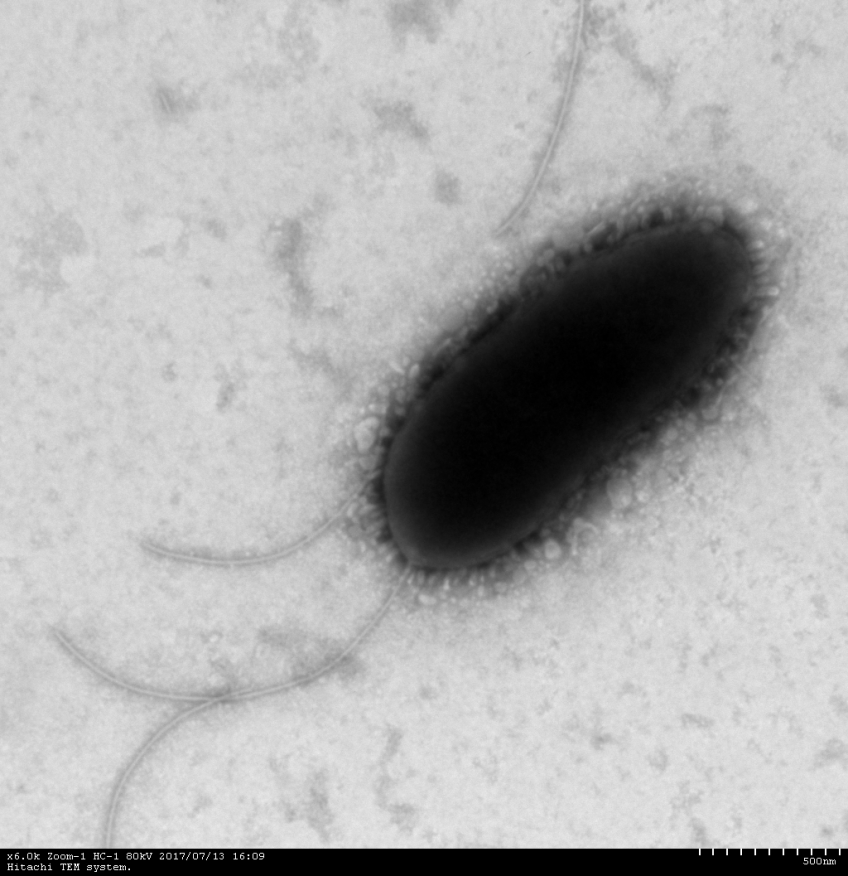


**(c)**

**Figure S2.** Morphological characteristics of strain W-7. (a) colony morphology (front); (b) colony morphology (back); (c) morphological characteristics observed under scanning electron microscope (6000×).

**Figure S3.** Antibiotic sensitivity of strain W-7. Strain W-7 was able to withstand 400 mg·mL^−1^ ampicillin; 50 mg·mL^−1^ kanamycin; and 200 mg·mL^−1^ gentamicin, streptomycin, and chloromycetin. Strain W-7 exhibited resistance against tetracycline up to 10 mg·mL^−1^.

**(a)**

**(b)**


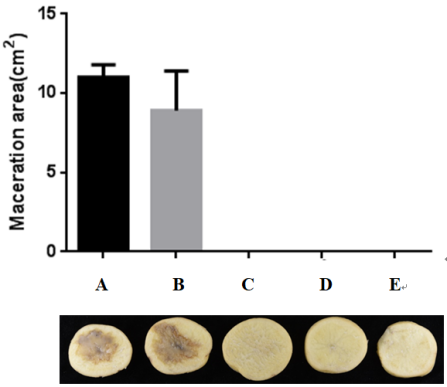

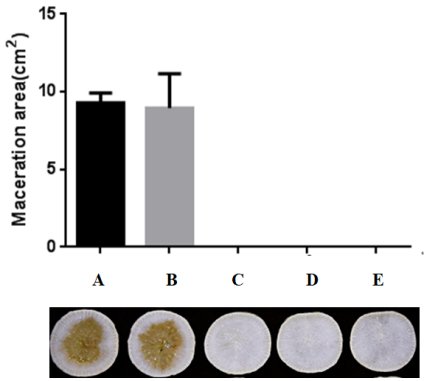


**(c)**

**(d)**


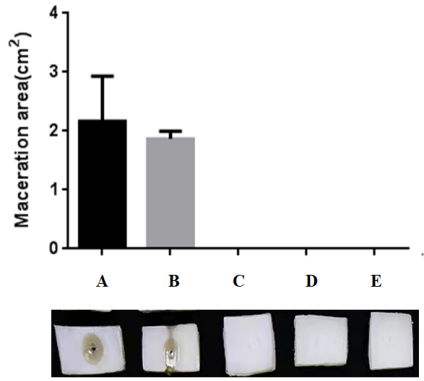

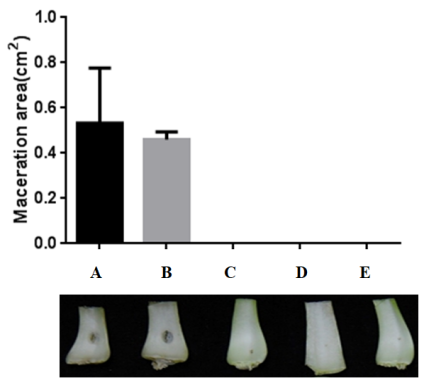


**Figure S4.** Test of strain W-7’s ability to attenuate maceration from soft-rot disease in different plant slices. (a) potato (*Solanum tuberosum* L.); (b) radish (*Raphanus sativus* L.); (c) Chinese cabbage (*Brassica pekinensis* (Lour.) Rupr. ); (d) pakchoi (*Brassica campestris*L. ssp. *chinensis*Makino). Panel A, EC1 alone on plant slices; Panel B, EC1 + *E. coli* DH5*α*; Panel C, EC1 + B23; Panel D, EC1 + agricultural streptomycin; Panel E, EC1+ W-7.

**A**

**B**

**Figure S5.** Comparison of the mass spectra of the degradation products with the authentic standard compounds of the National Institute of Standards and Technology (NIST, USA) library database. A: *N*-hexanoyl-L-homoserine lactone; B: *N*-cyclohexyl-propanamide. In each figure, the top is the spectrum of the sample, and the bottom is the spectrum of the standard compound in the NIST library database.


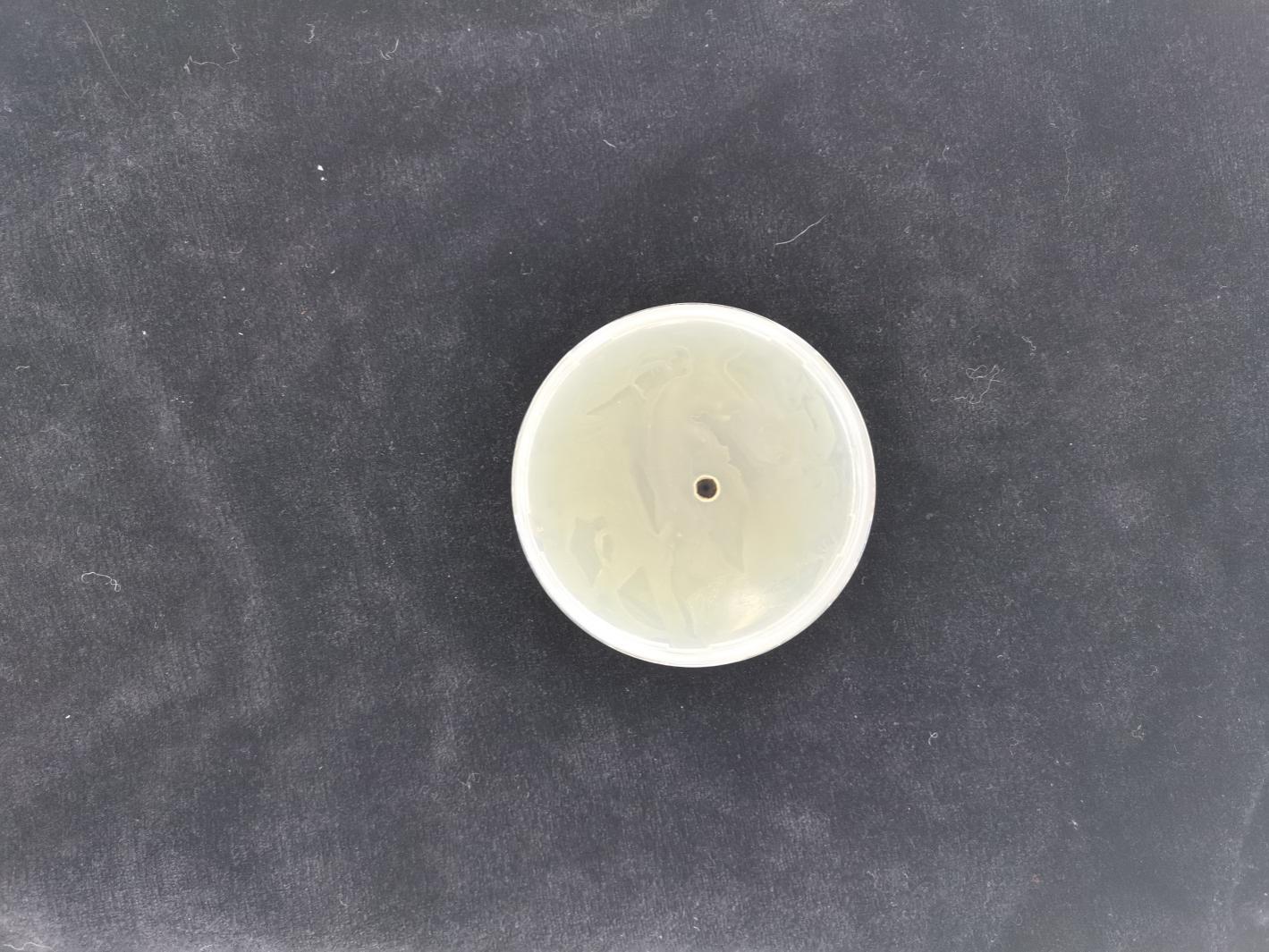

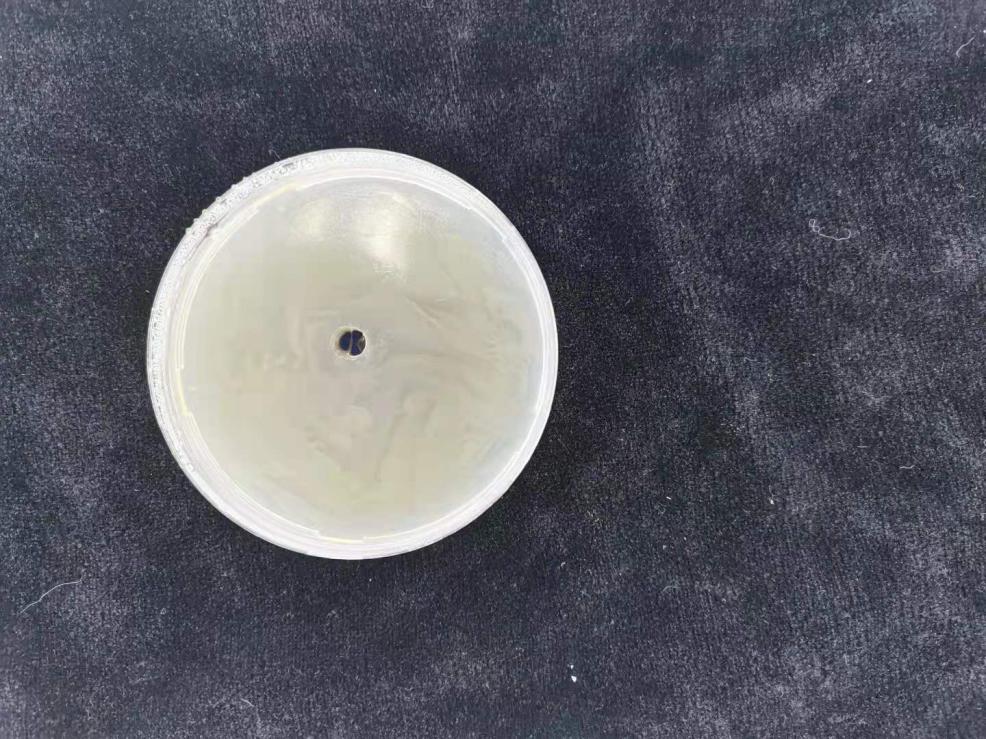


**B**

**A**


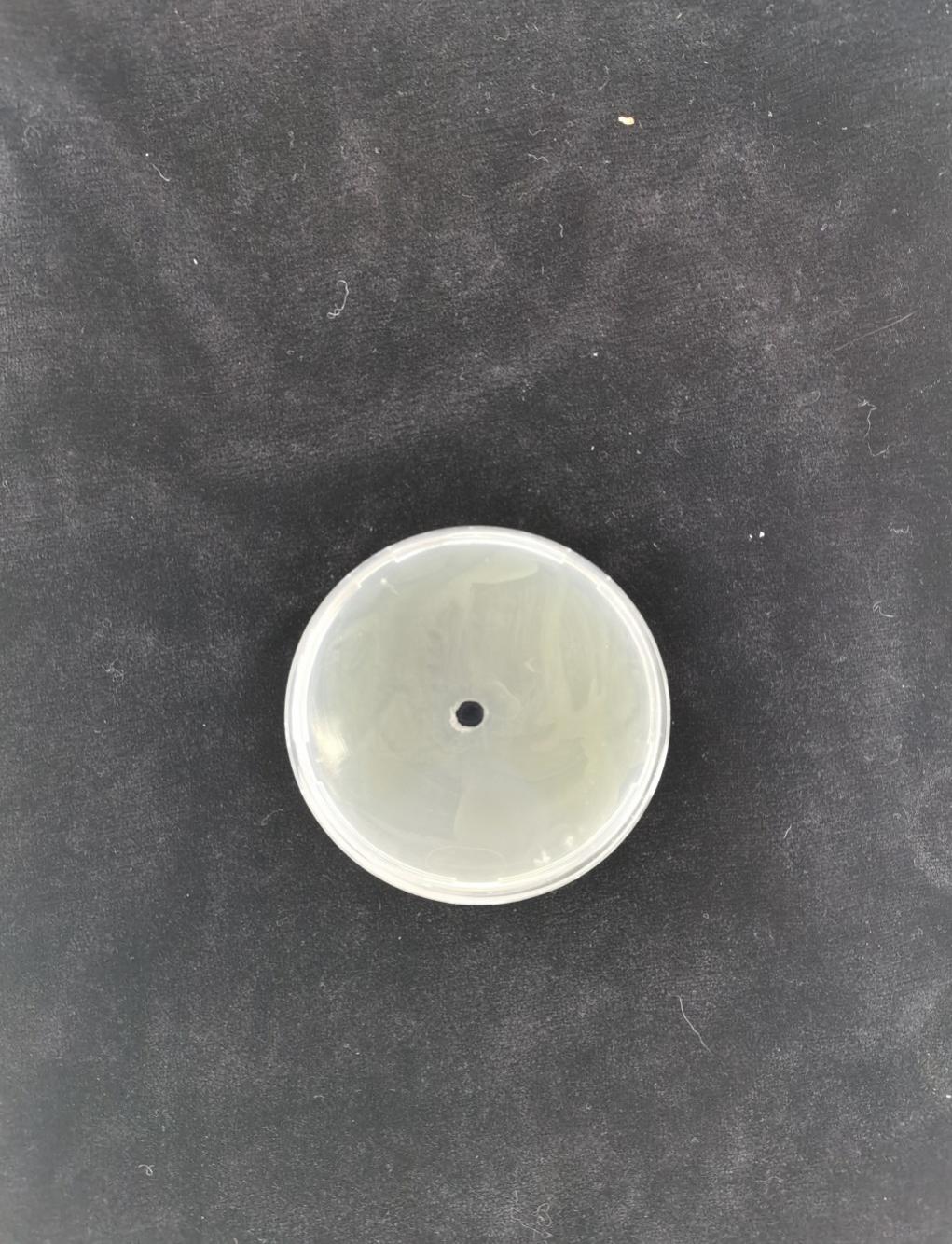

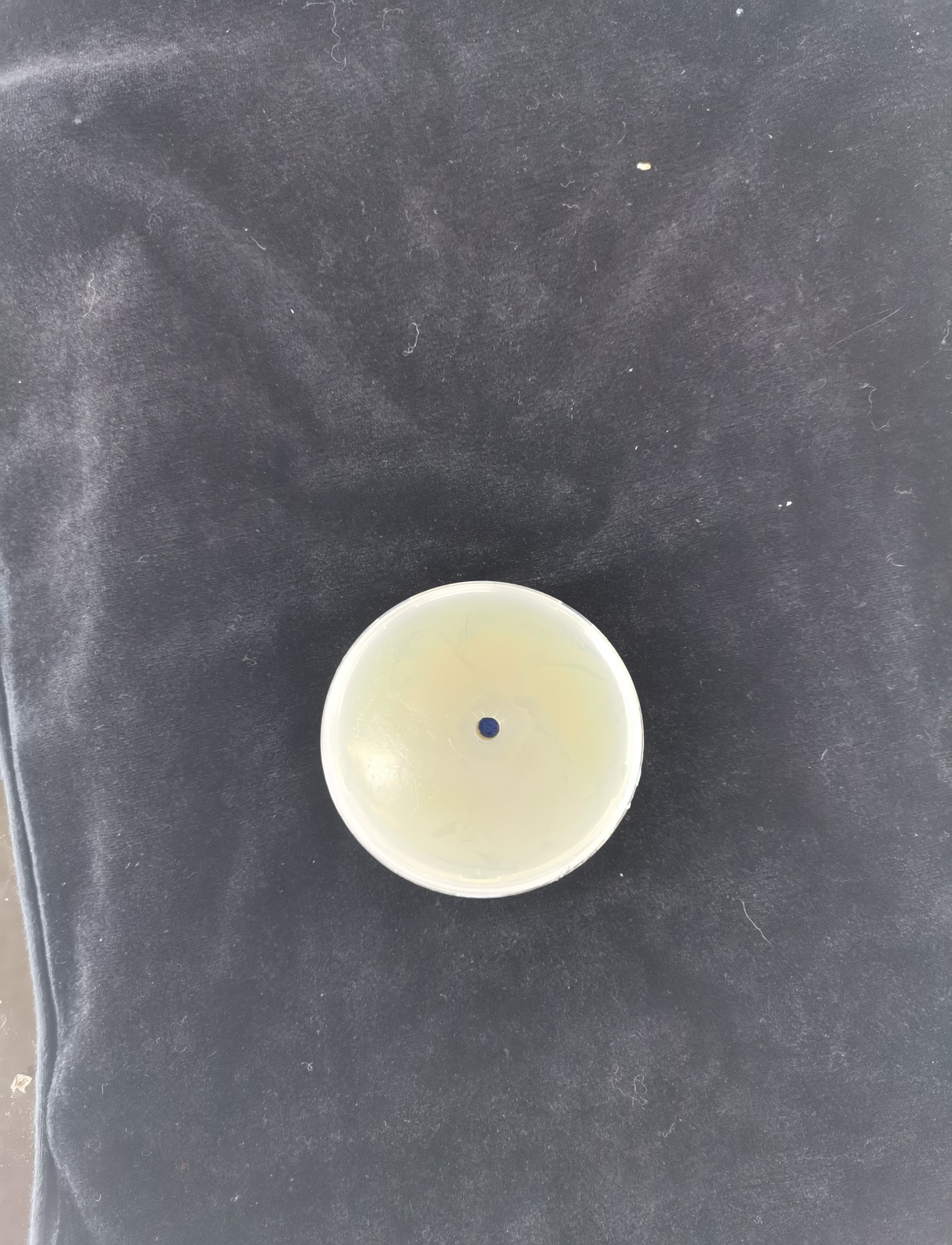


**D**

**C**

**Figure S6.** Antagonism test between *Dickeya zeae* strain EC1 and strain W-7. A: bacterial suspension of strain W-7; B: metabolites of strain W-7; C: methyl alcohol; D: sterile water. The results showed that no inhibition zone was occurred when strain W-7 and pathogen EC1 grew together.

**a**

**A B C D**


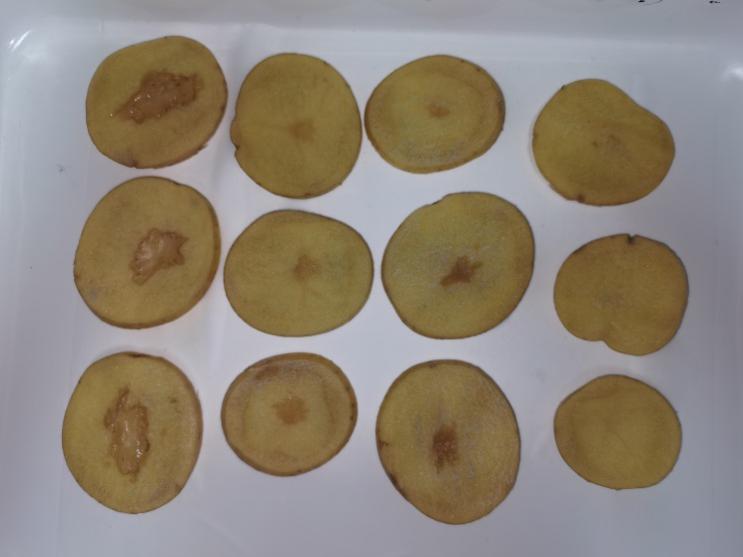


**b**

**(2)**

**(1)**

**Figure S7.** Preliminary biocontrol test of crude enzymes of strain W-7 against EC1. (a) Panel A: EC1 alone on plant slices; Panel B: EC1 + Extracellular enzyme; Panel C: EC1 + Intracellular enzyme; Panel D: Sterile water. (b) Maceration area (1) and maceration tissue (2) in each treatment. Statistical analysis was performed by one way ANOVA of Duncan method, and different letters indicate significant differences (*p* < 0.05) between treatments.

**CK A B**


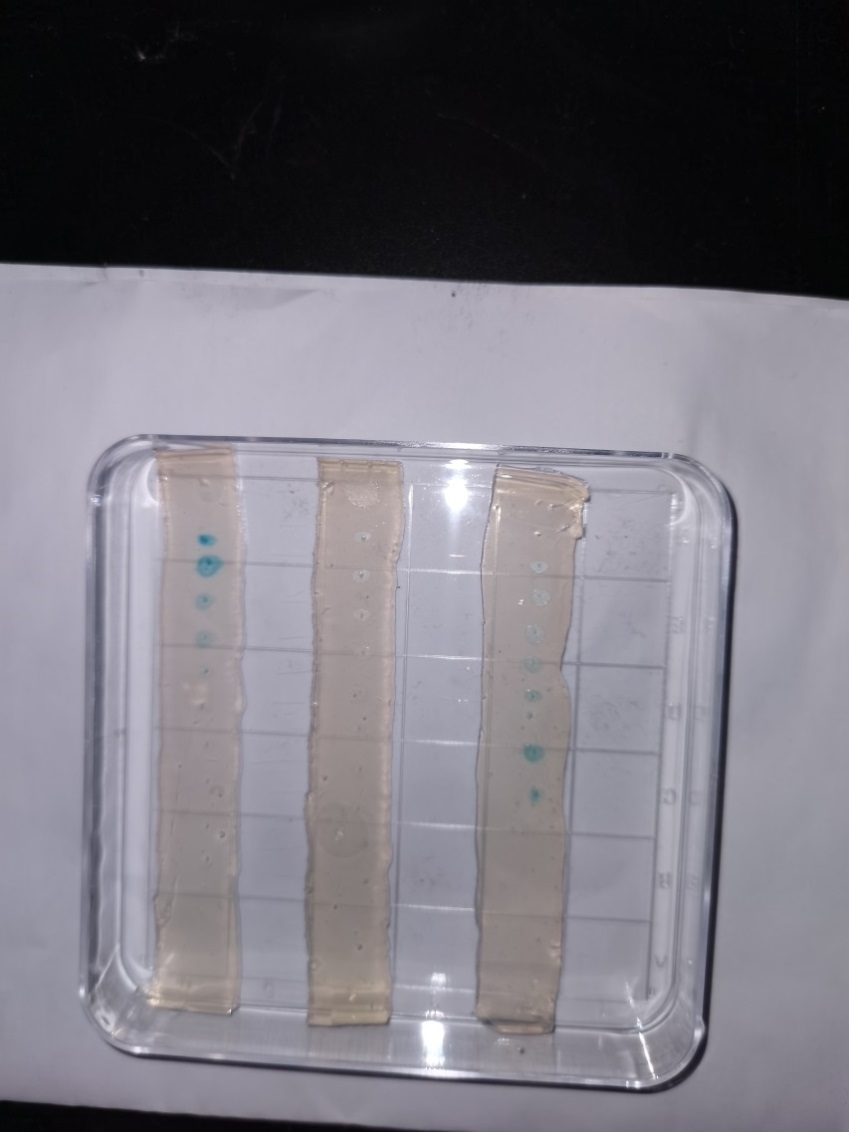


**Figure S8.** Re-lactonization assay. CK: OdDHL; A: strain W-7 + OdDHL; B: strain W-7 + OdDHL + 0.2 M HCl. The results showed that the OdDHL was degraded by strain W-7 (A) and reconstructed after acidification (B).

A B C

**
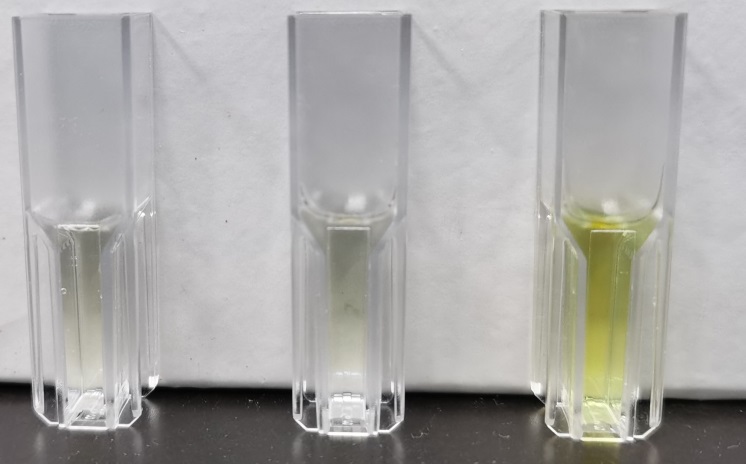
**

**Figure S9.** Acylase activity test. A: negative control, distilled water; B: strain W-7; C: positive control, *N*-acetyltransferase. Acylase can catalyze the transfer of the acetyl group of acetyl-CoA to butanol and, at the same time, reduce 5,5'-dithiobis-(2-nitrobenzoic acid) (DTNB) to generate 2-nitro-5-thiobenzoic acid (TNB). The TNB compound appears as yellow, with an absorption peak at 412 nm. The acylase activity test for strain W-7 was negative.

**Table S1** Physio-biochemical characteristics of strain W-7.

| Characteristics | Results | Characteristics | Results |
| --- | --- | --- | --- |
| Gram staining | Negative | Anaerobic test | Negative |
| Hydrogen sulfide | Negative | Catalase | Positive |
| Gelatin liquefaction | Negative | Oxidase | Positive |
| Haemolysis assay | Negative | Fluorochrome | Positive |
| Starch hydrolysis | Negative | Nitrate reduction | Positive |
| D-Maltose | Negative | Dextrin | Negative |
| Stachyose | Negative | D-Trehalose | Negative |
| Gentiobiose | Negative | Sucrose | Negative |
| D-Cellobiose | Negative | D-Turanose | Negative |
| D-Raffinose | Negative | α-D-Lactose | Negative |
| β-Methyl-D-  Glucoside | Negative | D-Melibiose | Negative |
| D-Salicin | Negative | *N*-Acetyl-D-  glucosamine | Negative |
| Sodium butyrate | Negative | N-Acetyl-D-  galactosamine | Negative |
| N-Acetyl  neuraminic acid | Negative | 1% NaCl | Positive |
| γ-Amino-butryric acid | Positive | 8% NaCl | Negative |
| α-D-Glucose | Positive | D-Mannose | Negative |
| D-Fructose | Negative | D-Galactose | Negative |
| 3-Methyl glucose | Negative | Nalidixic acid | Positive |
| Lithium chloride | Negative | L-Rhamnose | Negative |
| Inosine | Negative | 1% Sodium lactate | Positive |
| α-Keto-butyric acid | Negative | Acetoacetic acid | Negative |
| D-Sorbitol | Negative | D-Mannitol | Negative |
| Citric acid | Positive | α-Keto-glutaric acid | Positive |
| D-Arabitol | Negative | myo-Inositol | Negative |
| Aztreonam | Positive | D-Glucose-6-PO_4_ | Negative |
| Sodium bromate | Negative | D-Aspartic acid | Negative |
| Propionic acid | Positive | Troleandomycin | Positive |
| L-Malic acid | Positive | Minocycline | Negative |
| Rifamycin SV | Positive | Glycyl-L-Proline | Negative |
| Potassium tellurite | Positive | L-Arginine | Positive |
| L-Lactic acid | Positive | L-Glutamic acid | Positive |
| L-Histidine | Negative | L-Pyroglutamic acid | Negative |
| Tetrazolium blue | Positive | Lincomycin | Positive |
| Tetrazolium violet | Positive | Niaproof 4 | Positive |
| Pectin | Negative | Acetic acid | Positive |
| D-Lactic acid  Methyl ester | Negative | D-Gluconic acid | Positive |
| Mucic acid | Negative | Quinic acid | Positive |
| D-Saccharic acid | Negative | Vancomycin | Positive |
